# A herbivore that manipulates plant defence

**DOI:** 10.1111/j.1461-0248.2010.01575.x

**Published:** 2011-03

**Authors:** Renato Almeida Sarmento, Felipe Lemos, Petra M Bleeker, Robert C Schuurink, Angelo Pallini, Maria Goreti Almeida Oliveira, Eraldo R Lima, Merijn Kant, Maurice W Sabelis, Arne Janssen

**Affiliations:** 1 IBED, Section Population Biology, University of AmsterdamAmsterdam, The Netherlands; 2 Department of Animal Biology, Section Entomology, Federal University of ViçosaViçosa, Brazil; 3 Graduate Programme in Plant Science, Federal University of TocantinsPO BOX 66, Gurupi, TO, Brazil; 4 SILS, Department of Plant Physiology, University of AmsterdamAmsterdam, The Netherlands; 5 Department of Biochemistry and Molecular Biology, Federal University of ViçosaViçosa, Brazil

**Keywords:** Defence suppression, induced plant defence, jasmonic acid, plant volatiles, plant–herbivore interaction, predatory mites, proteinase inhibitors, salicylic acid, spider mites, *Tetranychus evansi*

## Abstract

Phytopathogens and herbivores induce plant defences. Whereas there is evidence that some pathogens suppress these defences by interfering with signalling pathways involved in the defence, such evidence is scarce for herbivores. We found that the invasive spider mite *Tetranychus evansi* suppresses the induction of the salicylic acid and jasmonic acid signalling routes involved in induced plant defences in tomato. This was reflected in the levels of inducible defence compounds, such as proteinase inhibitors, which in mite-infested plants were reduced to even lower levels than the constitutive levels in herbivore-free plants. Additionally, the spider mite suppressed the release of inducible volatiles, which are implicated in plant defence. Consequently, the mites performed much better on previously attacked plants than on non-attacked plants. These findings provide a new perspective on plant–herbivore interactions, plant protection and plant resistance to invasive species.

## Introduction

Plant defences can be induced by antagonists such as pathogens (Dangl & Jones 2001) and herbivores (Walling 2000). The molecular mechanisms involved in these defences are being elucidated rapidly (Kessler & Baldwin 2002; De Vos *et al.* 2005; Kant & Baldwin 2007; Pieterse & Dicke 2007). Induced plant defences critically depend on the detection and recognition of their attackers (Wu & Baldwin 2009), and one strategy for pathogens and herbivores to circumvent defences is to avoid being detected and feed stealthily (Walling 2008). The complex biochemical pathways involved in induced plant defence (Walling 2000) offer another way to elude defences; such pathways may be vulnerable to manipulation by pathogens (Nomura *et al.* 2006; McDowell & Simon 2008) and herbivores (Pieterse & Dicke 2007; Zarate *et al.* 2007). As a result of this manipulation, attacked plants may become even better resources for the pathogens and herbivores than non-damaged plants. Although such herbivore-induced susceptibility has often been suggested (Karban & Baldwin 1997), there is surprisingly little evidence for it. Earlier studies (for references, see Karban & Baldwin 1997) show increased herbivore attack of previously damaged plants, but the previous damage was often artificial and most of these studies did not measure herbivore performance, but preference.

Recently, some more convincing examples have been published. For example, glucose oxidase in caterpillar saliva reduces induced defences (Musser *et al.* 2002; Bede *et al.* 2006), and attacks by Colorado potato beetles suppress transcription of genes encoding for proteinase inhibitors (PI) involved in plant defence (Lawrence *et al.* 2008). None of these examples, however, has shown the effects of defence suppression on herbivore performance, and it has been suggested that suppression could even benefit the plant rather than the herbivore (Kahl *et al.* 2000). Other studies show that plants become more susceptible to attacks by herbivores after previous attacks by other species of herbivores (Sauge *et al.* 2006; Poelman *et al.* 2008). In these cases, trade-offs between defence types against different herbivore species may be involved (via so-called antagonistic cross-talk between signalling pathways involved in plant defence, Thaler 1999; see Bruessow *et al.* 2010 for an example of negative cross-talk within the same species). Herbivores may also vector plant pathogens, thereby inducing plant defences against pathogens, which in turn negatively affect the defences against herbivores (Belliure *et al.* 2005). In conclusion, examples of herbivore attacks that increase plant quality for conspecifics are scarce and the effects reported so far are small (Gonzales *et al.* 2002; Sauge *et al.* 2006; Bruessow *et al.* 2010). Thus, there is still little evidence for manipulation of plant defences by herbivores.

Recently, we showed that one population of a generalist herbivore, the spider mite *Tetranychus urticae*, harbours variation in its ability to suppress the induced defences of tomato plants to lower levels (Kant *et al.* 2008). This suppression had a small, but significant, positive effect on the fitness of non-suppressing mites residing on the same leaflet (Kant *et al.* 2008). Here, we report on a closely related mite species, *Tetranychus evansi*, which reduces induced tomato defences so severely that these drop even below house-keeping levels. Both *T. evansi* and *T. urticae* attack and eventually kill tomato plants, not because of high per capita consumption rates of individual mites, but due to their high population growth rates. Until the 1970s, the distribution of *T. evansi* was restricted to South America, where it attacks solanaceous plants, including tomato but then spread to Africa and Mediterranean Europe, where it now poses a serious threat to tomato crops, as it cannot be controlled by commercially available predators. We evaluated the performance of *T. evansi* on plants previously attacked by conspecifics and by *T. urticae* and the effect of these two species on induced plant defences. Specifically, we consider direct plant defences (i.e. defences that directly affect the performance of herbivores) and indirect defences (i.e. the attraction of natural enemies of the herbivores through the production of volatiles). We investigated the involvement of the two main signal-transduction pathways involved in plant defences.

## Material and methods

### Rearing methods

Tomato seeds (*Solanum lycopersicum* var Santa Clara I-5300) were sown in trays in a commercial substrate composed of vermiculite and organic fertilizer, and kept inside mite-proof screen cages in a greenhouse. Plants (21 days old) were transferred to plastic pots (2 L) that contained a mixture of soil plus bovine manure (3 : 1) and fertilizer (4-14-8 N-P-K). Tomato plants were further grown in mite-proof screen cages in a greenhouse until they were 45 days old and had at least four completely developed leaves. Subsequently, plants were used either for the experiments or for spider mite rearing.

Spider mites (*T. evansi* and *T. urticae*) were obtained in 2002 from naturally infested tomato plants of the same variety mentioned above, in a greenhouse at the Federal University of Viçosa, Brazil. Both species were cultured on detached tomato leaves, of which the petiole was inserted in a PVC tube with water to maintain leaf turgor. Tubes with infested leaves were kept in PVC trays filled with detergent and water (1 : 25, v/v), which served to prevent mite escapes and invasion of mites and other arthropods. The mass culture was maintained at 25 ± 3 °C, 70–90% relative humidity (RH) and 12 h light.

A culture of the predatory mite *Phytoseiulus longipes* was started in 2006 with individuals that were provided by Dr Gilberto de Moraes (USP São Paulo, Brazil). Colonies were maintained at 28 ± 2 °C; 80 ± 10% RH; 12 h light, supplied with detached tomato leaves from the *T. evansi* culture. A culture of another predatory mite species, *Phytoseiulus macropilis*, was started with specimens collected from lima bean plants infested with two-spotted spider mites in a greenhouse at the Federal University of Viçosa, Brazil. The mites were subsequently reared on detached tomato leaves infested with *T. urticae*, under identical conditions as above.

### Performance

Adult female spider mites produce 50–70% of their body weight in eggs per day, whereas juveniles increase much < 50% in weight per day. Hence, conversion of food is highest in adult females, and the rate of oviposition is so strongly related to population growth rate that it is a reasonable stand-in measure for fitness in local populations (Sabelis 1991). Furthermore, inferior host plant quality results in lower oviposition rates in spider mites (Li *et al.* 2002a). For this reason, we measured oviposition rate and adult female survival as follows. Two leaves of four randomly selected tomato plants of 45 days old were infested for 7 days with 200 adult female *T. urticae* or *T. evansi*, while the other leaves were kept clean. These numbers are within a realistic range for plants attacked by either of these two species (see Results). Both mite species were collected from tomato plants at the Federal University of Viçosa. The non-infested control plants (*N* = 8) had the same age. Insect glue (Cola Entomológica; Bio-Controle, São Paulo, Brazil) was applied to the petioles of the leaves on which mites were released to prevent mites from moving to other leaves. Control plants from the same batch and the same age were also treated with glue. Plants were kept inside mite-proof screen cages in a greenhouse for 7 days. After removal of mites, web and eggs, 10 leaf discs (20 mm Ø) were made from one damaged and one non-damaged leaf of each infested plant and of corresponding leaves of non-infested control plants. Leaf discs were kept individually in Petri dishes (8 cm Ø) on top of wet cotton wool. One randomly selected adult female *T. evansi*, 2 days old since turning adult, was placed on each disc. After 2 days (28 ± 2 °C; 70 ± 10% RH; 14 h light), discs were replaced with new discs from the other, similarly treated leaf of the same plant, on which the mites were kept for another 2 days. Leaf discs were checked every day for survival of the mites and oviposition rates were corrected for mortality. Differences in mean oviposition rates per plant among treatments were tested with a linear mixed effects (lme) model on ln(x + 1) transformed numbers of eggs with female and plant as a random factor. Differences in survival and the number of mites escaped were compared among treatments with a generalized linear model (GLM) with a binomial error distribution. Note that the experiments with *T. urticae* and *T. evansi*, as well as those on PI and volatile analysis could not be carried out at the same time for logistical reasons. As it was impossible to maintain constant greenhouse conditions, plant quality may have varied with time. Treatments could therefore only be compared with their controls within the same experiment.

Juvenile development was measured on similarly prepared leaf discs (10 discs of four plants of each treatment and of eight control plants), on which one egg from a cohort of eggs was placed. Survival and development were monitored twice per day until all individuals had either died or matured. Data were subjected to a Kaplan–Meier survival analysis (Hosmer & Lemeshow 1999). Log-rank tests were used for pairwise planned comparisons (Hosmer & Lemeshow 1999).

### Proteinase inhibitor assays

Proteinase inhibitors are involved in induced plant defence. They are induced by herbivore attacks and hamper the action of digestive proteinases present in the herbivore gut (Koiwa *et al.* 1997), including those of spider mites (Li *et al.* 2002a; Kant *et al.* 2004). Proteinase inhibitor activity was measured in non-damaged and damaged leaves of plants treated as in the oviposition experiments as well as in non-damaged plants (*N* = 4 plants). An amount of 600 mg of leaf tissue was frozen in liquid nitrogen and stored at −80 °C. Subsequently, it was ground in liquid nitrogen and homogenized in 1000 μL extraction buffer (0.1 m Tris-HCl buffer, pH 8.2 and 20 mm CaCl_2_; 1 : 3 w/v), centrifuged at 17 200 ***g*** (30 min, 4 °C). Fifty μL trypsin (4.7 × 10^−5^ m) was mixed with 50 μL of the supernatant and 500 μL extraction buffer (0.1 m Tris-HCl buffer, pH 8.2 and 20 mm CaCl_2_), and was incubated at room temperature for 5 min. Controls consisted of 500 μL extraction buffer and 50 μL of trypsin (4.7 × 10^−5^ m). A 500 μL aliquot of the mixture was added to 500 μL extraction buffer and 500 μL Na-Benzoyl-D,L-arginine 4-nitroanilide hydrochloride (1.2 mm). Trypsin activity was monitored with a spectrophotometer (410 nm). The difference between the absorbance measured at 150 and 60 s was used to determine trypsin activity. Measurements were performed in triplicate per sample, and were converted to mg trypsin inhibited per gram of protein (Kant *et al.* 2004) and were corrected for the dilution (Kakade *et al.* 1974). Differences among treatments were tested with a lme model with replicate as a random factor.

### Gene expression

The induction of PI genes such as *WIPI-II* and downstream protein activity depends on the jasmonic acid (JA) signal-transduction pathway, which is mainly activated after attacks by herbivores and necrotrophic pathogens (Walling 2000; De Vos *et al.* 2005; Zarate *et al.* 2007). However, attacks of herbivores like whiteflies, aphids and spider mites can also activate the salicylic acid (SA) pathway (Walling 2000; Kant *et al.* 2004; De Vos *et al.* 2005). To verify which defensive pathways were affected by *T. evansi*, we measured the expression of the genes *WIPI-II*, *PR-P6* and *GGPS1*. The induction of *WIPI-II* is both dependent on the biosynthesis (Li *et al.* 2002b) and on perception of JA (Li *et al.* 2004) and is a direct indicator of the JA response. The tomato *PR-P6* gene is a pathogenesis-related protein, induced by SA (Ament *et al.* 2006; van Schie *et al.* 2007b) and a direct indicator of the SA response. *GGPS1* produces the precursor for the volatile homoterpene (E,E)-4,8,12-trimethyltridecae-1,3,7,11-tetraene (TMTT), which is attractive to predators of spider mites (Kant *et al.* 2004). This gene is induced by JA, but a basal level of SA is required for its induction (Ament *et al.* 2006).

Expression analyses were performed on non-damaged leaves and damaged leaves of plants (45 days) infested (7 days) with 200 *T. urticae* or 200 *T. evansi* females as above. Leaflets were cut in small pieces and immediately stored in RNALater® (Applied Biosystems, Foster City, CA, USA) according to the manufacturer's instructions. Gene specific quantitative RT-PCR primers were designed for the target genes as well as an endogenous control gene Rub 1 conjugating enzyme (*RCE1*). *WIPI-II* fw: GAC AAG GTA CTA GTA ATC AAT TAT CC; *WIPI-II* rv: GGG CAT ATC CCG AAC CAA GA. *PR-P6* fw: GTA CTG CAT CTT CTT GTT TCC A; *PR-P6* rv: TAG ATA AGT GCT TGA TGT GCC. *RCE* fw: AGT TGC GTC TTC ATA AAG ATA TAA G; *RCE* rv: GAA CGT AAA TGT GCC ACC CAT A. *GGPS1* fw: GGC AGA TTG TGG ACT TGG CGA; *GGPS1* rv: CTC ATT CGC TCC ACA TCA ACC (van Schie *et al.* 2007a). Total RNA was extracted using the hot phenol method. Purity and integrity was checked by NanoDrop (ND-1000, ThermoFisher, Waltham, MA, USA) analyses and agarose-gel electrophoresis respectively. Two μg DNase I (Ambion, Turbo DNA-free kit #AM1907, Austin, TX, USA)-treated RNA was used for reverse transcription and first strand cDNA synthesis with MuLV-RT reverse transcriptase and samples were treated with RNAse H according to the manufacturer's instructions (Fermentas Int. Burlington, Canada, RevertAid kit EP0441). For analysis, the cDNA equivalent of 10 ng total RNA was used as a template and mixed with 2× SYBR Green PCR Master Mix, ROX reference dye and 300 nm of each primer and dispersed as 20 μL on a 96-well optical reaction plate (Applied Biosystems). Specificity of the reaction was verified by dissociation analysis. Gene expression of *RCE1* was used to normalize and correct for variance in quality of RNA and quantity of input cDNA. Primer pair efficiencies were estimated by analysis of the amplification curves and the average efficiency of all reactions on a plate was used in calculations. Three biological replicates and two technical replicates were analysed individually. Relative expression levels were log-transformed and analysed with manova. anovas were used for each gene separately, followed by *post hoc* comparisons among treatments.

### Volatile analysis

The attack of plants by herbivores induces the production of specific plant volatiles, which are implicated to play a role in plant defence. These volatiles can repel herbivores (Pallini *et al.* 1997; Kessler & Baldwin 2001), are thought to be involved in within-plant signalling (Frost *et al.* 2007) and can attract natural enemies of herbivores (Dicke *et al.* 1990; Turlings *et al.* 1990; Thaler 1999; Kessler & Baldwin 2001). In tomato, *GGPS1* codes for the enzyme geranylgeranyl diphosphate synthase, which synthesizes the precursor for the volatile TMTT, which is attractive to predators of spider mites (Dicke *et al.* 1990). We therefore analysed the volatiles produced by tomato plants attacked by either *T. evansi*, *T. urticae* or no herbivores. Plants (45 days old) were infested with mites as above and groups of three plants were used as odour sources. Three leaves from each group of plants were put in a glass jar, wrapped in black paper to avoid light penetration. Charcoal-filtered air was led over the leaves using a vacuum pump. Volatiles were collected for 24 h at an airflow of 150 mL min^−1^ on 50 mg of Super Q (80/100 mesh; Alltech, Deerfield, IL, USA) connected to the outlet of the jar. Before use, the filter was rinsed with 2 mL of methanol and HPLC grade *n*-hexane (Sigma-Aldrich, St Louis, MO, USA). Volatiles were washed off with 0.4 mL of HPLC grade *n*-hexane (Sigma-Aldrich), collected in sealed glass capillary tubes and stored at −20 °C. Volatiles were analysed as described in Bleeker *et al.* (2009). All datasets were processed with the Metalign software package (Lommen 2009) for automated baseline correction, mass spectra extraction and subsequent spectral data alignment, with settings advised for the Leco Pegasus TOF-MS (St. Joseph, MI, USA), using auto scaling on the total signal. The metabolites of these mass peaks were subsequently identified in the original GC-MS (Gas Chromatography–Mass Spectrometry) chromatograms. Differences in log-transformed volatile production between treatments and controls were tested with lme models with replicate as a random factor.

### Host plant selection

Plants were infested with spider mites as described above (Volatile analysis). Non-damaged plants of the same age were used as control odour sources. The attraction of individual spider mites and predatory mites to the odours emanating from these plants was assessed using a Y-tube olfactometer (Pallini *et al.* 1997). Prior to testing, adult female *T. evansi* were starved for 24 h (28 ± 2 °C; 70 ± 10% RH); predators for 3 h. Mites were tested individually in the olfactometer as described in Pallini *et al.* (1997). Data were analysed with a GLM with a Poisson error distribution (Crawley 2007).

### Number of dispersers

Populations of *T. evansi* and *T. urticae* grow fast and in the absence of predators eventually over-exploit their host plant, after which the mites disperse to new plants. As result of this metapopulation structure (Ellner *et al.* 2001), the per capita oviposition rate or fecundity are not adequate stand-in fitness measures, but rather the number of dispersers produced per patch, as shown by Metz & Gyllenberg (2001). We therefore measured the final numbers of *T. evansi* and *T. urticae* on tomato plants at the moment the plant was overexploited, assuming that this is proportional to the number of dispersers. Tomato plants were infested with three 2-day-old mated females of *T. evansi* or *T. urticae* with a fine brush. Plants were maintained inside a climate room (28 ± 2 °C, 70 ± 10% RH and 12 h of light) during the whole experimental period. Adult female mites of both mite species on the plants were counted every 3 days until the plants died of overexploitation by the mites. Each treatment was replicated four times (i.e. four different plants). Log-transformed densities of spider mites were analysed using lme models, to correct for repeated measures. All statistical analyses were performed in R (R development Core Team 2006).

## Results

### Performance

**Figure 1:**
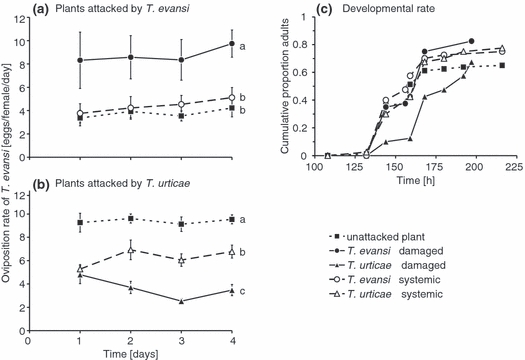
Performance of *Tetranychus evansi*. Mean (± SEM, four plants, 10 mites per plant) oviposition of *T. evansi* on tomato leaves that were previously damaged by conspecific mites (a, closed circles, solid line) or *Tetranychus urticae* (b, closed triangles, solid line), on non-infested leaves of infested plants (systemic, open circles and triangles and interrupted line) and leaves from non-damaged control plants (eight plants, 10 mites per plant, closed squares and dotted line). The effect of treatment was significant (linear mixed effects model; a: *F*_2,13_ = 9.85, *P* = 0.0025; b: *F*_2,13_ = 44.6, *P* < 0.0001). Curves with different letters within each panel differ significantly. The experiments with *T. urticae* and *T. evansi* were not carried out at the same time for logistical reasons. As it was impossible to maintain constant greenhouse conditions, plant quality will have varied with time. Treatments can therefore only be compared with their controls within the same experiment (i.e. within the same panel). (c) Developmental rate and juvenile survival of *T. evansi* (in h, from egg to adult) on tomato leaves treated as in a and b. The curves show the fraction of mites of the initial cohort of 40 eggs per treatment that developed into adults. Error bars are left out for reasons of clarity.

*Tetranychus evansi* had a significantly higher oviposition rate on tomato leaves that were previously attacked by conspecifics than on leaves of non-damaged tomato plants or on systemic leaves (i.e. non-attacked leaves from previously attacked plants) (Fig. 1a). Mortality of adult females during the oviposition experiment was significantly lower on leaves previously attacked by *T. evansi* than on non-attacked leaves (GLM, χ^2^_1,10_ = 6.27, *P* = 0.012), but was not significantly different from that on systemic leaves (χ^2^_1,10_ = 2.42, *P* = 0.12) (average mortality ± SE: non-attacked: 0.389 ± 0.098; systemic: 0.331 ± 0.164; damaged: 0.167 ± 0.047).

The oviposition rate of *T. evansi* on tomato leaves previously attacked by *T. urticae* was lower than on non-damaged leaves (Fig. 1b), suggesting that *T. evansi* is sensitive to defences induced by *T. urticae*. These results also confirm that the inducible defences in our tomato variety were intact. The mortality of adult *T. evansi* was significantly higher on leaves damaged by *T. urticae* than on systemic leaves (χ^2^_2,14_ = 8.1224, *P* = 0.0044) and than on non-attacked leaves (χ^2^_2,14_ = 28.9, *P* < 0.0001) (non-attacked: 0.025 ± 0.016; systemic: 0.125 ± 0.048; damaged: 0.40 ± 0.041). Notice that the experiments with plants attacked by *T. urticae* and *T. evansi* were not carried out at the same time for logistical reasons, and plant quality may have varied with time. Oviposition rates and mortality on leaves attacked by *T. evansi* and *T. urticae* can therefore not be compared directly, but only with their respective controls.

There was no effect of plant treatment on juvenile survival of *T. evansi* (Fig. 1c; χ^2^_4_ = 1.6, *P* = 0.804), but there was a significant effect on developmental rate (Fig. 1c; χ^2^_4_ = 20.1, *P* = 0.0005). This was caused by the developmental rate of juveniles on leaf discs from plants previously attacked by *T. urticae* being lower than in all other treatments (Fig. 1c, all *P* values < 0.05).

### Proteinase inhibitor assays

**Figure 2:**
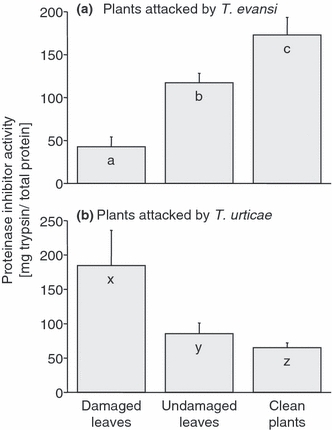
Proteinase inhibitor activity. Mean (+ SEM, *n* = 4 plants) proteinase inhibitor (PI) activity in leaves damaged by *Tetranychus evansi* (a) or *Tetranychus urticae* (b) and non-damaged leaves from similarly damaged plants (systemic) and leaves of non-damaged plants. The effect of treatment was significant (anova on log-transformed relative activity: *F*_3,12_ = 25.6, *P* < 0.0001). Within each panel, bars labelled with different letters are significantly different (Tukey HSD). The experiments with *T. urticae* and *T. evansi* were not carried out at the same time for logistical reasons. As it was impossible to maintain constant greenhouse conditions, plant quality will have varied with time. Treatments can therefore only be compared with their controls within the same experiment (i.e. within the same panel).

Levels of PI activity in leaves attacked by *T. evansi* were less than half that of activity levels in leaves of non-damaged plants (Fig. 2). PI activity in systemic leaves was higher than in damaged leaves (Fig. 2), but somewhat lower than in non-damaged leaves, suggesting that the effect was, at least partially, systemic. Attacks by *T. urticae* increased PI activity (Fig. 2), as reported before (Kant *et al.* 2004), showing that induced plant defence through increased PI activity was intact in the tomato plants.

### Gene expression

**Figure 3:**
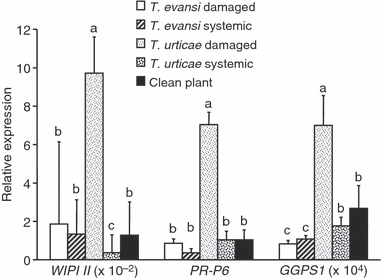
Expression of genes regulated by jasmonic acid (JA) or salicylic acid (SA). Mean expression levels normalized to *RCE1* (+ SEM, *n* = 3, two technical replicates) of *WIPI-II*, *PR-P6* and *GGPS1* in leaves attacked by *Tetranychus evansi* (white bars), systemic leaves of the same plants (crosshatched bars), leaves attacked by *Tetranychus urticae* (light dotted bars), systemic leaves (dark dotted bars) and leaves of non-attacked plants (black bars). The effect of treatment was significant (manova: Pillai-Bartlett statistic = 1.41, d.f. = 4,13, *P* = 0.0060), as was the effect on each gene separately (anova: *WIPI-II*: *F*_4,13_ = 4.78, *P* = 0.014; *PR-P6*: *F*_4,13_ = 4.67, *P* = 0.015; *GGPS1*: *F*_4,13_ = 6.13, *P* = 0.005). Bars with different letters within each gene differ significantly.

*Tetranychus evansi* did not up-regulate *WIPI-II* (JA pathway) or *PR-P6* (SA pathway) (Fig. 3). In contrast, leaves attacked by *T. urticae* showed strongly increased expression of these genes (Fig. 3), confirming earlier results (Kant *et al.* 2004) and demonstrating that normal establishment of induced defences is hampered by *T. evansi*. The *GGPS1* gene is involved in indirect plant defence in tomato (Kant *et al.* 2004). Tomato leaves attacked by *T. evansi* showed down-regulation of *GGPS1* (Fig. 3). Leaves attacked by *T. urticae* showed the normal up-regulation of *GGPS1* relative to un-attacked plants, in agreement with earlier reports (Kant *et al.* 2004) (Fig. 3).

### Volatile analysis

**Figure 4:**
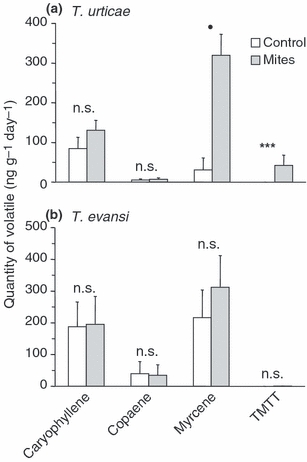
Production of volatile plant compounds involved in indirect plant defence. Shown are mean (+ SEM) volatile profiles of plants. (a) Plants (*n* = 3) attacked by *Tetranychus urticae* (grey bars) produced more (E,E)-4,8,12-trimethyltridecae-1,3,7,11-tetraene (TMTT) than non-damaged plants (white bars) (lme, *F*_1,2_ = 34.8, *P* = 0.0275, ***) and marginally significantly more myrcene (*F*_1,2_ = 7.75, *P* = 0.108, •). (b) Plants (*n* = 5) attacked by *Tetranychus evansi* (grey bars) and non-damaged plants (white bars) did not differ significantly (lme, all *P* values > 0.17).

Two volatiles were emitted in higher amounts by leaves under attack by *T. urticae* than by control leaves (Fig. 4a): TMTT, which is known to be induced by *T. urticae* (Kant *et al.* 2004), and β-myrcene, which also increases upon *T. urticae* infestation, but not always significantly (Kant *et al.* 2004). The two other volatiles shown in Fig. 4, β-caryophyllene and α-copaene, are constitutively produced by tomato plants and not induced by *T. urticae* (Kant *et al.* 2004). In contrast, the emission of volatiles of plants attacked by *T. evansi* was not different from that of non-damaged plants (Fig. 4b). Univariate statistical analysis, an integral part of Metalign, was used for mass peak selection, with 95% significance, to compare the means of each individual aligned mass peak from infested leaves with the controls. Metalign did not identify any fragments that were significantly induced or reduced in leaves attacked by *T. evansi* compared with control leaves (all *P* values > 0.10). Hence, *T. evansi* does not induce the production of the same volatiles as *T. urticae*.

### Host plant selection

**Figure 5:**
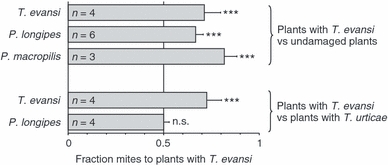
Attraction of mites by odours of tomato plants attacked by *Tetranychus evansi*. Bars represent the fraction of mites choosing for the odours of plants attacked by *T. evansi*, a fraction of 0.5 would indicate no preference. Fractions were averaged (+ SEM) over replicates (numbers given inside the bars), carried out using different sets of three plants and different groups of 20 mites. The spider mite *T. evansi* (top bar) and the predatory mites *Phytoseiulus longipes* and *Phytoseiulus macropilis* (second and third bar) preferred odours of plants attacked by *T. evansi* to odours of non-damaged plants. Moreover, *T. evansi* preferred odours of plants attacked by *T. evansi* to odours of plants attacked by *Tetranychus urticae* (fourth bar) and *P. longipes* did not show preference for either odour (lower bar). ***GLM: *P* < 0.001; n.s.: GLM: not significant.

The lack of induction of the production of volatiles by *T. evansi* was further tested through the response of *T. evansi* and two species of predatory mite to the volatiles emitted by plants attacked by *T. evansi* and *T. urticae*. Predatory mites are important natural enemies of spider mites; they are often used to control them and they are attracted to plant volatiles induced by spider mites (Dicke *et al.* 1990), including tomato plants (Kant *et al.* 2004). The two species used here, *P. longipes* and *P. macropilis*, co-occur with *T. evansi* in South America, and *P. longipes*, is considered a potential biological control agent of *T. evansi* (Furtado *et al.* 2007). All three mite species significantly preferred odours of plants with *T. evansi* to those of non-damaged plants, suggesting that the plant–herbivore complex did release attractive odours (Fig. 5). Moreover, *T. evansi* had a clear preference for odours of plants attacked by conspecifics when offered together with odours of plants with *T. urticae* (Fig. 5), confirming that the volatiles emanating from plants with these two closely related spider mites are different. In contrast, *P. longipes* showed no preference for odours of plants with *T. urticae* or *T. evansi* (Fig. 5).

### Number of dispersers

**Figure 6:**
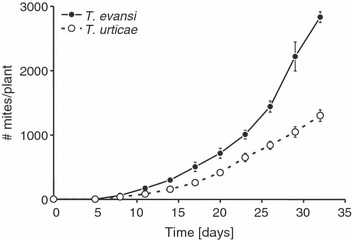
Population growth rates of *Tetranychus evansi* and *Tetranychus urticae* on intact tomato plants. Shown are mean densities (+ SEM, *n* = 4) of adult female *T. evansi* (closed symbols) and *T. urticae* (open symbols) until the plants were overexploited. Through time, the densities of *T. evansi* were significantly higher than those of *T. urticae* (linear mixed effects model, χ^2^ = 116.7, d.f. = 1, *P* < 0.0001).

The effect of reduced plant defences caused by *T. evansi* did not result in more rapid over-exploitation of the host plant compared with the defence-inducing *T. urticae*. In contrast, it ultimately gave rise to higher numbers of mites per plant, hence, higher numbers of dispersers (Fig. 6), which is a stand-in measure for fitness in populations with a metapopulation structure (Metz & Gyllenberg 2001).

## Discussion

We show that the phytophagous mite *T. evansi* had a higher oviposition rate and a higher adult survival on plants that were previously attacked by conspecifics than on non-damaged plants. These results show that *T. evansi* has a higher performance on plants that were previously attacked by conspecifics. In contrast, *T. evansi* had a lower oviposition rate, lower juvenile and adult survival and a lower juvenile developmental rate on plants that were previously attacked by its congener *T. urticae*. This corresponds to what is normally observed as the effect of induced plant defence; herbivore performance is lower on previously attacked plants than on non-damaged plants (Walling 2000).

The higher oviposition rate of *T. evansi* on previously attacked plants coincided with these plants having lower PI activity. It is known that tomato *def-1* mutants, which are deficient in wound-induced JA accumulation and expression of downstream target genes, do not show increased PI activity upon herbivore attack. However, these *def-1* plants show similar house-keeping levels of PI activity as non-damaged wild-type plants (Li *et al.* 2002a), indicating that constitutive, house-keeping, PI levels are regulated differently than JA-dependent induced PI levels (O'Donnell *et al.* 2003). As the PI activity in plants damaged by *T. evansi* was significantly lower than that in non-damaged plants (Fig. 2), this shows that, unlike other herbivores (Musser *et al.* 2002; Bede *et al.* 2006; Kant *et al.* 2008; Lawrence *et al.* 2008; Walling 2008), *T. evansi* does not just prevent induction of plant defences but reduces house-keeping levels of defence-related plant constituents below the levels in non-attacked plants. This suggests that the mite reduces constitutive plant defences as well.

The low activity of PI in leaves previously attacked by *T. evansi*, in turn, coincided with lack of up-regulation of *WIPI-II*, a gene that is dependent on the JA defensive pathway, one of the two main signalling routes involved in plant defence in tomato. Likewise, *PR-P6*, a marker gene of the other main signalling route, the SA pathway, was also not up-regulated by *T. evansi* damage. The fact that marker genes from neither of the two pathways were up-regulated shows that the lack of induction of either of the two pathways was not caused by negative cross-talk between the SA and JA signalling pathways (Thaler 1999; Bruessow *et al.* 2010).

*GGPS1* was also not up-regulated in plants attacked by *T. evansi*, in agreement with the lack of TMTT emission by these plants. Counter to our expectation, *T. evansi* and two species of predatory mite were more attracted to plants attacked by *T. evansi* than to non-damaged plants in an olfactometer (Fig. 5), showing that more volatiles or more volatile compounds emanated from the infested plants. The fact that *T. evansi* prefers odours of plants attacked by conspecifics to plants attacked by *T. urticae* confirms our finding that the volatiles emanating from plants attacked by these two species differ, but evidently, *P. longipes* did not discriminate between these volatiles (Fig. 5). Apparently, the volatiles that we identified are not of key importance for the attraction of these natural enemies (see D'Alessandro & Turlings 2005 for a similar case for parasitic wasps). The inducible plant volatiles found here are known to be attractive for other species of predatory mite (Dicke *et al.* 1990), but may also be involved in other defensive functions (Frost *et al.* 2007).

The high oviposition rate of *T. evansi* on tomato plants resulted in a high population growth rate on tomato plants (the local population growth rate estimated from Fig. 6 is 0.300 ± 0.009 day^−1^), which is the highest recorded for spider mites at the prevailing temperature (Sabelis 1991). The estimated growth rate of *T. urticae* (Fig. 6, 0.262 ± 0.005) closely coincided with the average intrinsic growth rate published for this species (0.269 ± 0.020) (Sabelis 1991), showing that our results with the strain of *T. urticae* and the variety of tomato tested do not differ from earlier reports. The growth rate of *T. urticae* is generally considered to be among the highest for spider mites, but it was significantly lower than that of *T. evansi* (*t*-test, *t*_6_ = 3.63, *P* = 0.012), which is striking because tomato is generally considered as a low quality food source for *T. urticae* (Chatzivasileiadis & Sabelis 1997; Agrawal *et al.* 2002). However, this high growth rate did not result in more rapid overexploitation (death) of the host plants, but rather, in higher population levels at the moment of overexploitation compared with *T. urticae*. This will result in higher numbers of mites dispersing from overexploited plants.

In conclusion, *T. evansi* seems to interfere with plant defences in a manner different from other herbivores. There is very limited evidence for defence suppression by herbivores (Musser *et al.* 2002; Bede *et al.* 2006; Zarate *et al.* 2007; Lawrence *et al.* 2008), and even less evidence that this suppression results in an increase in plant quality for the suppressing herbivore. Rather than merely suppressing defences downstream of the induced response, *T. evansi* apparently also interferes with components of house-keeping mechanisms that maintain constitutive *WIPI* transcript levels and PI activity levels in non-damaged plants. This results in suppressed plants being better food than induced plants and herbivore-free control plants.

The last decades, much of the research on plant–herbivore interactions was focused on plant defence, but such defences are bound to trigger selection for counter-adaptations in herbivores (Karban & Agrawal 2002). The down-regulation of tomato defences by *T. evansi* may be an example of this. More research into the molecular mechanisms underlying plant defence suppression is needed to develop crops able to counter manipulation by herbivores. Such research will further our understanding of the evolution of plant–herbivore interactions and the interplay of plant defences with herbivore strategies to counter these defences (Karban & Agrawal 2002).
